# Supplementary material for: Visualization and Interpretation of Multivariate Associations with Disease Risk Markers and Disease Risk—The Triplot
Source: Metabolites. 2019 Jul 6;9(7):133. doi: 10.3390/metabo9070133 (PMC6680952; doi:10.3390/metabo9070133)
Supplement: Supplementary file 1 [file metabolites-09-00133-s001.pdf]

Supplementary Materials

Visualization and interpretation of multivariate associations with disease risk markers and disease risk – The triplot

Tessa Schillemans[1#], Lin Shi[2#], Xin Liu[3], Agneta Åkesson[1], Rikard Landberg[2,4], Carl Brunius[2*]

[1] Cardiovascular and Nutritional Epidemiology, Institute of Environmental Medicine, Karolinska Institute, Stockholm, Sweden; tessa.schillemans@ki.se; agneta.akesson@ki.se

[2] Department of Biology and Biological Engineering, Chalmers University of Technology, Gothenburg, Sweden; shlin@chalmers.se; carl.brunius@chalmers.se; rikard.landberg@chalmers.se

[3] Department of Epidemiology and Biostatistics, School of Public Health, Xi'an Jiaotong University Health Science Center, Xi'an, Shaanxi, China; xinliu@xjtu.edu.cn

[4] Department of Public Health and Clinical Medicine, Umeå, University, Sweden; rikard.landberg@chalmers.se

* Correspondence: carl.brunius@chalmers.se; Tel: +46-70-4834385

\# Tessa Schillemans and Lin Shi contributed equally to this work.

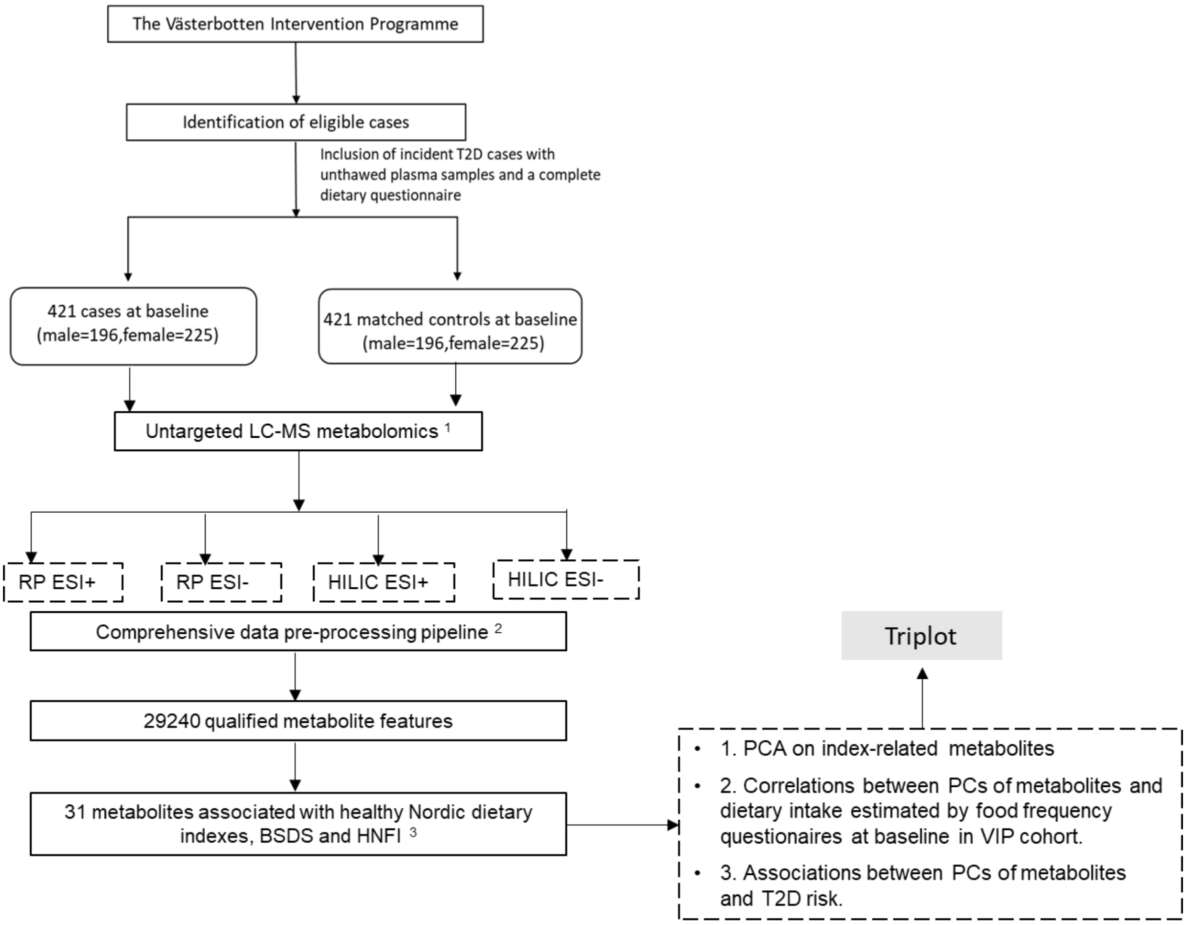

Figure S1. Overall workflow for generation of the data in the original *HealthyNordicDiet* Study. Plasma samples from 421 matched T2D case-control pairs at baseline were analyzed by untargeted liquid chromatography-mass spectrometry (LC-MS) metabolomics. Samples were analyzed on reverse phase C18 (RP) and hydrophilic interaction chromatography (HILIC) columns, with both positive (ESI+) and negative (ESI−) electrospray ionization. Raw data acquired in each analytical batch were processed using the open source R package "XCMS", followed by signal drift correction using the R package "batchCorr". In total 31 metabolites associated with a priori-defined healthy Nordic dietary indexes, i.e. the Baltic Sea Diet Score (BSDS) and Healthy Nordic Food Index (HNFI), were identified using a validated random forest-based variable selection and partial Spearman correlation adjusted for case-control status, age at blood draw, gender, BMI, smoking status, education and physical activity. The BSDS included 9 food components: fruits, vegetables, whole grains, fish, red and processed meat, low-fat milk products, ratio of polyunsaturated fatty acids to the sum of saturated fat and trans-fatty acids (fat ratio), estimated total alcohol intake and total fat. The HNFI included 6 food items: fish, cabbage, rye bread, oatmeal, apples/pears, and carrots. The triplot was generated using synthetic data with similar internal covariance structure as the original data. In the original study, the principal component analysis (PCA) (R package "psych") was performed on the 31 index-related metabolites. Partial Spearman rank correlations were calculated between PCA scores and both indices as well as 50 individual food items reported in food frequency questionnaires with <0.5% missing values, after adjustment for age, gender, case status, smoking status, education, and physical activity. Only those correlations significant in the original data were used to generate the triplot in the manuscript and tutorial. The associations between PCA scores and risk of T2D were investigated using conditional logistic regression, adjusting for lifestyle-related factors, i.e., smoking status, education, physical activity at diet assessment, and daily energy intake.

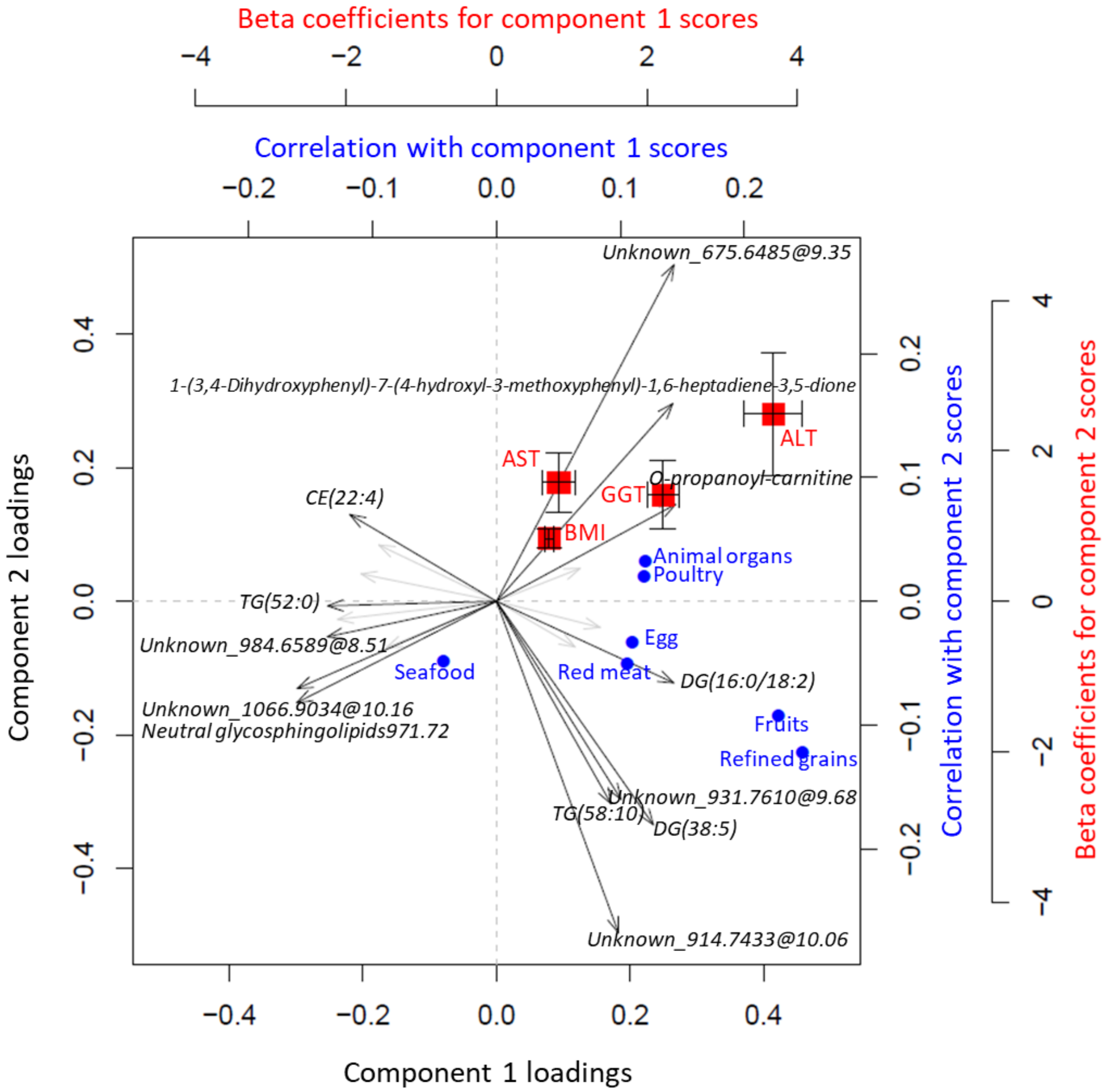

Figure S2. A PLS-based triplot visualizing the intercorrelations between plasma metabolites predicting BMI, dietary intake variables and metabolic traits, adjusting for age and gender using the synthetic 'CAMP' data. Correlations between latent variables (LV) and dietary intakes were calculated using partial Spearman method, adjusted for age and gender. Associations of LVs with metabolic traits were calculated using linear regression adjusted for age and gender. Only metabolite feature loadings >0.25, significant correlations as well as correlations with animal derived foods and metabolic traits with strongest associations are visualized. ALT: alanine aminotransferase; AST, aspartate aminotransferase; GGT: gamma-glutamyltransferase.

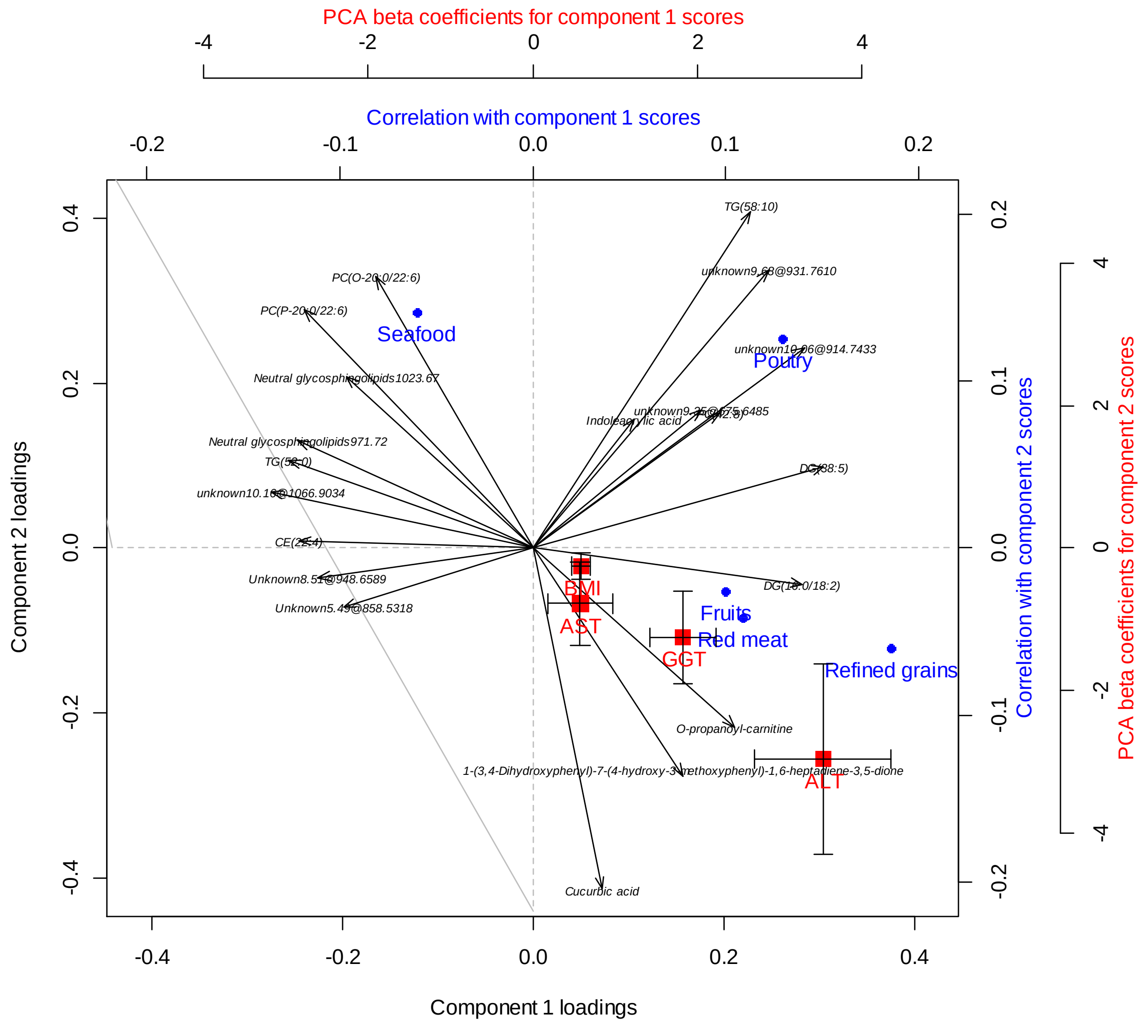

Figure S3. A PCA-based triplot visualizing the intercorrelations between plasma metabolites predicting BMI, dietary intake variables and metabolic traits, adjusting for age and gender using the authentic 'CAMP' data. Correlations between latent variables (LV) and dietary intakes were calculated using partial Spearman method, adjusted for age and gender. Associations of LVs with metabolic traits were calculated using linear regression adjusted for age and gender. Only metabolite feature loadings >0.2, significant correlations as well as correlations with animal derived foods and metabolic traits with strongest associations are visualized. ALT: alanine aminotransferase; AST, aspartate aminotransferase; GGT: gamma-glutamyltransferase.
